# A comparison of long-term survivors and short-term survivors with glioblastoma, subventricular zone involvement: a predictive factor for survival?

**DOI:** 10.1186/1748-717X-9-95

**Published:** 2014-04-23

**Authors:** Sebastian Adeberg, Tilman Bostel, Laila König, Thomas Welzel, Juergen Debus, Stephanie E Combs

**Affiliations:** 1 Department of Radiation Oncology, University Hospital of Heidelberg, Im Neuenheimer Feld 400, 69120 Heidelberg, Germany; 2 Heidelberg Ion Therapy Center (HIT), Im Neuenheimer Feld 450, 69120 Heidelberg, Germany

**Keywords:** Glioblastoma, Subventricular zone, Long-term survival, Prognostic factors

## Abstract

**Objective:**

Long-term survival is rare in patients with glioblastoma (GBM). We set out to determine prognostic factors for patients with favorable and poor prognosis in regard of tumor localization to the subventricular zone (SZV).

**Methods:**

We reviewed the clinical records, pre-operative and post-operative MRI imaging of 50 LTS long-term survivors (LTS) (> 3 years) and 50 short-term survivors (STS) (< 1 year) with glioblastoma. These groups were matched for clinical characteristics being consistently associated with prolonged or shortened survival. All patients had undergone initial surgery or biopsy to confirm GBM diagnosis followed by radio- or chemoradiotherapy.

**Results:**

LTS had a median progression-free survival PFS of 25, 4 months (2, 3–97, 8 months) and overall-survival (OS) of 55, 9 months (38, 2-98, 6 months) compared to STS who had a significantly lower PFS of 4, 2 months (1, 4–10, 2 months) and OS of 6, 6 months (2, 2–11, 6 months) (each p < 0,001).

Survival analysis showed that age under 60 years (p < 0,001), total resection status (p < 0,001) and tumor localization without SVZ contact (p = 0,05) were significant factors for prolonged survival.

**Conclusion:**

Our findings underline that survival in GBM patients is heterogeneous and influenced by multiple factors. This study confirms that tumor location with regard to the SVZ is significantly associated with survival.

## Introduction

Glioblastoma (GBM) are the most common primary brain tumors in adults with poor survival rates of approximately 15 months after advanced chemoradiotherapy [1].

Even though multimodal therapy approaches improved, prognosis changed little over the last decades [2]. Up to date it is known that only about 2% of all GBM patients survive longer than 36 months, a number that is likely to increase in the near future due to therapeutic improvements [3,4]. Interestingly individual patient survival is heterogeneous. The search to identify prognostic parameters for shortened or prolonged survival is therefore currently ongoing [5,6].

Numerous patient characteristics like age, gender, performance status and tumor localization have been identified as potential prognostic factors [3,7]. Furthermore, molecular markers like MGMT hypermethylation and IDH1 mutation seem to play a growing role as predictors for prognosis and therapeutic response in glioblastoma patients [8-10]. GBM LTS and STS have not been studied in detail concerning tumor location in reference to the SVZ. Recent studies demonstrated that the heterogeneity in patient survival and recurrence patterns of patients with GBM may be related to neuronal stem cells, located in the SVZ [10,11].

From a group of 909 patients, we selected LTS (> 36 months) and STS (< 12 months) to focus on outcome in these patients and to determine prognostic factors. Thus, 100 patients were included into the present study, we analyzed LTS and STS to validate prognostic factors for patients diagnosed with GBM.

## Patients and methods

### Patient population

We identified 100 patients who matched our criteria of LTS (survival over 36 months from the date of initial diagnosis) or STS (survival under 12 months from the date of initial diagnosis) with primary GBM [7,12-14]. We included 50 patients with LTS and 50 patients with STS who have been treated with radiotherapy or chemoradiation between January 2004 and August 2013 at the Department of Radiation Oncology/University Hospital Heidelberg, Germany. Treatment decisions were made according to the current treatment standard at the time, respecting patient-individual factors such as age or performance status. Patient characteristics of both groups are shown in Table 1.

**Table 1:** Patient characteristics and results of short- and long-term survivors with glioblastoma

**Patient characteristics of STS and LTS**		
	**STS; n = 50**	**LTS; n = 5O**
Age (years)	64, 8	51, 9
< 60 years	16 (32)	35 (60)
> 60 years	34 (68)	15 (30)
< 70 years	34 (68)	43 (86)
> 70 years	16 (32)	7 (14)
Male	33 (66)	26 (52)
Female	17 (34)	24 (48)
Tumor location		
Right hemisphere	16 (32)	24 (48)
Left hemisphere	24 (48)	23 (46)
Both hemispheres	10 (20)	3 (6)
Location close to the ventricle (< 10 mm)	44 (88)	33 (68, 8)
Location distant to the ventricle (≥ 10 mm)	6 (12)	15 (31, 3)
Involvement of the ventricle system	30 (60)	18 (39, 1)
Multifocal disease	12 (24)	7 (15, 2)
Subependymal spread	15 (30)	5 (10, 9)
Extent of resection		
Biopsy	26 (52)	5 (10, 4)
Total resection	7 (14)	22 (45,8)
Partial resection	17 (34)	22 (45,8)
Ventricle opening during surgery	8 (16)	17 (34,7)
Classification (Lim et al.)		
Type I	18 (36)	21 (43,8)
Type II	16 (32)	6 (12, 5)
Type llI	9 (18)	15 (31, 3)
Type IV	7 (14)	6 (125)
MGMT hypermethyation	4 (36, 4)	12 (70, 6)
Radiotherapy within a study	23 (46)	25 (50)
GD (Gy)	56, 8	45, 1
RT as Re-irradiation	3 (6)	15 (30)
Radiochemotherapy	35 (70)	38 (76)
Temozolomide	32 (64)	35 (70)

Numbers in brackets represent percentages and refer to the absolute values in front.

There were 33 (66%) male and 17 (34%) female patients in the STS group with a median age of 64, 8 years (ranging from 42, 5 to 77, 5 years) at the time of radiotherapy. 26 (52%) males and 24 (48%) females were included in the LTS group with a mean age of 51, 9 years (ranging from 20, 3 to 73, 3 years). 23 (46%) STS and 25 (50%) LTS received radiotherapy within a study.

We reviewed the clinical, hospital course records as well as pre-operative and post-operative MRI imaging. Data evaluation was performed according to institutional guidelines.

Neuropathological diagnosis was determined according to the most recent WHO classification system [15].

Patients were followed prospectively at the center initially 6 weeks after treatment and in 3 months intervals in the following period until progress. No patient was lost to follow-up which included contrast enhancing MRI imaging. Preoperative images and follow-up examinations until progress or death were assessed.

### Radiotherapy/chemoradiotherapy

For treatment planning, patients were fixed with custom-made mask fixation, computed tomography and MRI imaging were proceeded. A median dose of 60, 0 Gy (range 12, 0–68, 0 Gy) was delivered. Target volume definition included the primary tumor region, including all contrast-enhancing lesions (CEL) on T1-weighted MRI and the T2-hyperintense region. A 2 to 3 cm safety margin for potential microscopic spread was added.

### Imaging

All patients underwent preoperative MRI, treatment planning CT and postoperative follow up MRI available on the clinical intern picture archiving and communication system (PACS).

Initial tumor localization and relapse localization were determined based on T1 weighted sequences on axial and coronal images. Images were assessed (T.W.) and reviewed (T.B.) by experienced radiological specialists. The following parameters were determined: localization of the primary lesion, shortest distance of the CEL to the ventricle system and subependymal spread. Multifocal disease was defined as the presence of > 1 independent CEL noncontiguous to the initial lesion. Involvement of the ventricle system was valued if the CEL contacted the lining of the ventricle. Accordingly tumors were classified as involving the cortex if the CEL contacted the cortex. Tumors were considered close to the ventricle if the shortest distance to a ventricle was ≤ 10 mm and considered close to the ventricle if the distance was > 10 mm. According to previously published findings [10], LTS and STS were classified in 4 different subtypes in reference to their location to the ventricle system.

### Statistical analysis

Analyses were performed using the software program SigmaPlot™ (Systat Software GmbH, Germany). Survival calculations were performed using Kaplan-Meier survival curves. Prognostic factors were compared using the Wilcoxon signed-rank test and the Mann–Whitney-U-Test. Correlation of patient characteristics in subtypes were determined using the Odds ratios, χ ^2^-test and corresponding 95% confidence intervals.

## Results

### Patient collective

We identified 50 STS and 50 LTS with primary GBM who were treated with chemoradiotherapy/radiotherapy over the 9 year study period. The median age of the STS was significantly higher with 64, 8 years (range, 42, 5-77, 5 years) compared to 51, 9 years (ranging from 20, 3-73, 3 years) in LTS (p < 0,001). The gender distribution in STS works with 2/3 in favor of the men (p = 0,22). In comparison the genders were spread almost evenly in the group of LTS (m = 52%; f = 48%).

### Treatment and pretreatments

A total of 24 STS (48%) underwent surgical resection as initial treatment, which was complete in 7 cases (29, 2%) and incomplete in 17 (70, 8%) cases. 26 patients (52%) underwent biopsy only. 44 LTS were initially treated with total (n = 22; 50%) or partial resection (n = 22; 50%). Gross total tumor resection was performed more frequent in LTS (OR: 5,002; 95% CI: 1,884 to 13,300; p < 0,001). In 5 cases (10, 4%) a biopsy was performed exclusively. Resection status of one LTS was not determinable with certainty. This patient was excluded in survival analysis with regards of resection status. In conclusion, STS had a higher rate of biopsies for diagnosis confirmation (OR = 9,533; 95% CI: 3,242 to 28,034, p < 0,001) and LTS received surgery more often (OR: 5,005; 95% CI: 1,884 to 13,300, p = 0,002). There was no significant difference of ventricle openings during surgery in STS (n = 8; 16%) and LTS (n = 17; 34, 7%) (OR = 0,359; CI 95%: 0,138 to 0,934) (Table 1).

Concomitant chemotherapy with temozolomide (TMZ) was applied to 32 (64%) STS and 38 LTS (76%). 3 (6%) STS and LTS each received combined therapy with a different agent (bevacizumab, cetuximab or temsirolimus). No significant difference was observed between the LTS and STS (OR = 0,737; 95% CI: 0,303 to 1,789).

3 (6%) STS and 15 (30%) LTS received radiotherapy as a re-irradiation. Tumors of long-term survivors (n = 12, 70, 6%) had a higher occurrence frequency of hypermethylated MGMT promoter status than in STS (n = 4; 36,4%).

Radiotherapy was performed as a re-irradiation in a significantly higher percentage in LTS with 30% (n = 15) compared to only 12% (n = 6) in STS (p = 0,05).

### Tumor localization

STS had a higher frequency (n = 10; 20%) of pretherapeutic bihemispheric tumor spread than LTS (n = 3; 6%) (p = 0,074). Apart from that, the aspect ratio was distributed uniformly.

**Figure 1:**
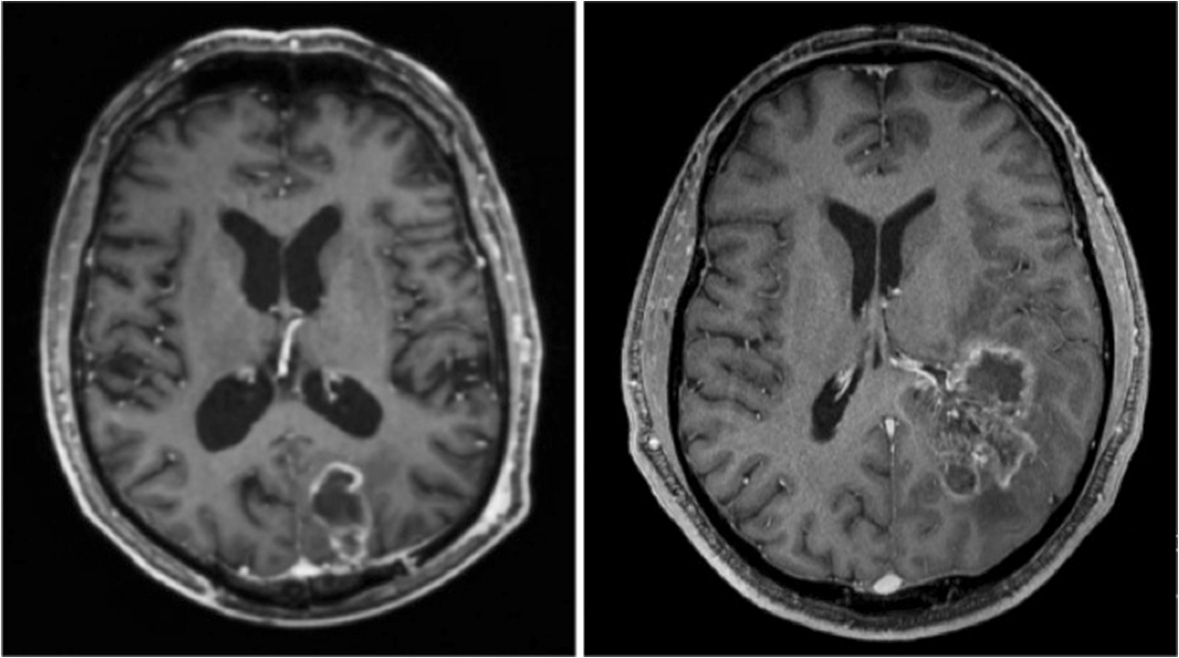
**Glioblastoma on T1-weighted, post-contrast magnetic resonance images on the axial view.** Tumor localization in the cortex without subventricular zone involvement (left). Glioblastoma with infiltration of the subventricular zone and subependymal spread (right).

Significantly more STS (n = 44; 88%) showed a tumor localization close to the ventricle system (≤ 10 mm) than LTS (n = 33, 68, 8%) (OR = 3,333; 95% CI: 1,168 to 9,514; p = 0,038). Accordingly more GBM in LTS (n = 15; 31, 3%) were located distant (> 10 mm) to the closest ventricle (p = 0,05). GBM of STS tended (p = 0,05) to infiltrate the SVZ more frequent (n = 34; 68%) compared to LTS (n = 27; 56, 3%). Conversely GBM of LTS were located more likely (n = 21; 43, 8%) in the cortex area or white matter not infiltrating the SVZ than STS (n = 16; 32%), as shown in Figure 1 (OR = 2,789; 95% CI: 1,156 to 6,731). In summary, ventricle involvement was more common in STS (n = 30; 60%) compared to the LTS group (n = 18; 39, 1%) (p = 0,66). There was no significant correlation between multifocal disease occurrence in both groups (p = 0,308). 15 STS (30%) who were diagnosed with GBM showed a subependymal tumor spread at the time of radiotherapy in contrast to only 5 LTS (10, 9%) (OR = 3,514; CI: 95%: 1,160 to 10,643; p = 0,04).

### Survival

**Figure 2:**
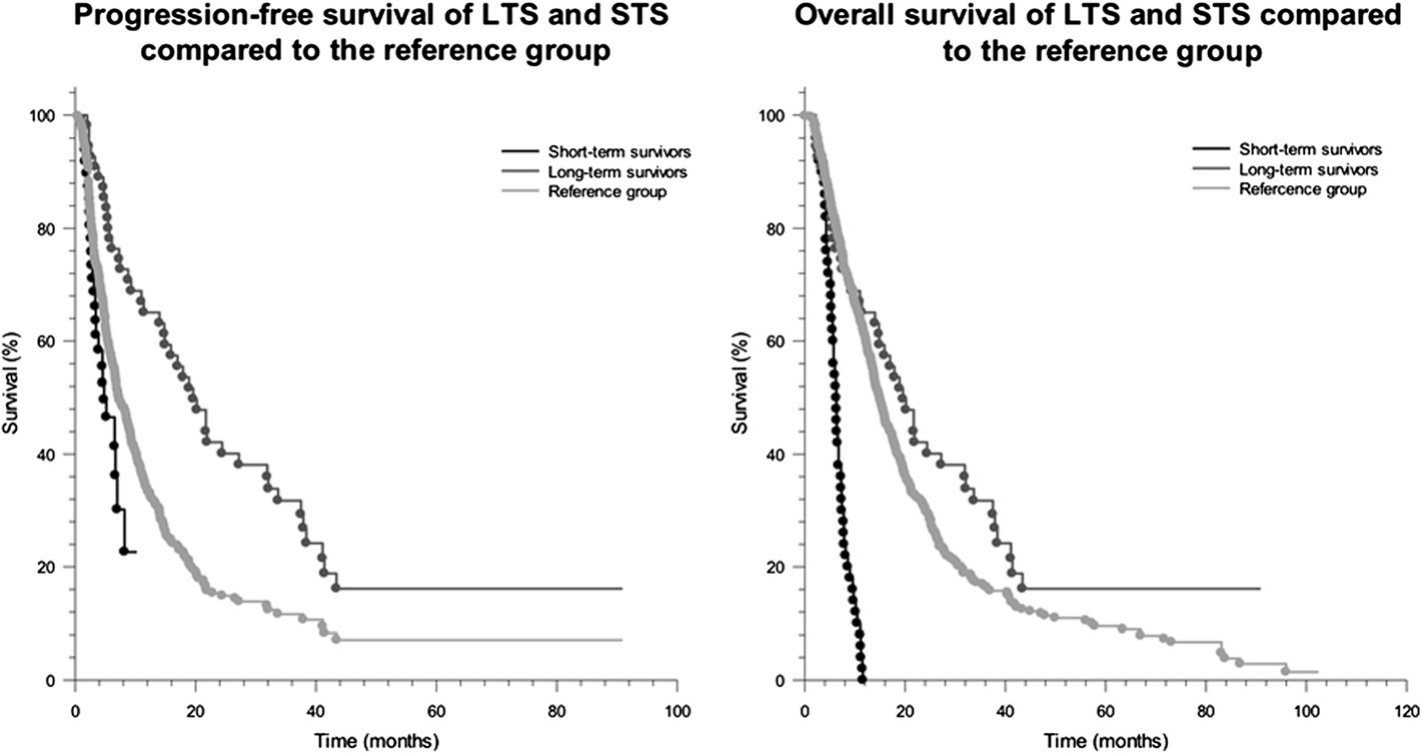
Progression-free survival (left) and overall survival (right) of short-term survivors, long-term survivors and a reference group of glioblastoma patients.

Long-term survivors had a significantly higher PFS with 25, 4 months (range: 2, 3–97, 8 months) and OS with 55, 9 months (range: 38, 2-98, 6 months) than the control GBM patients, that we have published previously with a PFS of 10, 4 months (±6, 7 months) and mean OS of 17, 7 months (±14, 8 months) (p < 0,001). Accordingly, STS showed a significantly lower PFS of 4, 2 months (range: 1, 4–10, 2 months) and OS of 6, 6 months (range: 2, 2–11, 6 months) than the control GBM group (p < 0,001) (Figure 2).

OS rates of LTS after complete resection with SVZ contact were significantly higher than in STS after complete resection with SVZ contact (86, 9 vs. 6, 3 months; p < 0,001). However LTS after partial resection with SVZ involvement had a significantly decreased OS compared to LTS after partial resection not involving the SVZ (41, 8 months vs. 83, 1 months; p < 0,001). Whereas LTS after complete tumor resection without SVZ contact did not show a significant survival advantage compared to LTS after complete resection with SVZ (48, 6 vs. 47, 1 months; p = 0,5). Keeping in mind that in LTS following complete tumor resection, 7 of 10 patients (70%) without SVZ contact and 5 of 10 patients (50%) with SVZ contact were still alive at the last follow up examination.

## Discussion

Our study shows that long-term survival in GBM patients is possible. It is known that only about 2, 2% of GBM patients survive longer than 3 years after initial diagnosis [3,16]. Preoperative clinical and radiological parameters for survival remain understudied, even though few predictive values like performance status and age have been determined in the past. Therefore we endeavored to identify clinical parameters for prolonged and shortened survival. Our idea was to correlate patient characteristics of both extremes, long-term survivors (OS > 36 months) on the one hand and short-term survivors (OS ≤ 12 months) on the other.

The analysis of age distribution showed that LTS had a significantly lower mean age (51, 9 years) than average GBM patients (59, 5 years) as well as STS (64, 8 years) (p < 0,001). This indicates that older age (> 60 years) is associated with decreased survival and vice versa. Our findings are consistent with previous published studies [12,17,18]. However, 7 LTS were aged over 70 years, implying that higher age per se does not exclude LTS and this should be taken into account when defining treatment algorithms for elderly patients.

Overall the incidence rate of glioblastoma is about 1, 4-1, 6 of male to female patients [19]. In our collective of LTS, the ratio was 1,1 and 1,9 in STS. These findings indicate that females are overrepresented in LTS and males in STS. Hence no statistical significance could be observed. Krex et al. also described a higher proportion of females, with a male/female ratio of 1,04 in their LTS-collective of 55 patients showing that female gender seems to be favorable for increased survival [12].

Besides basic patient characteristics, recent researches have shown that tumor localization in reference to the SVZ and potential stem-like glioblastoma cells is a predictive factor for survival [7] (*). Interestingly our data showed a higher rate of glioblastoma localization in cortical areas or white marrow without SVZ contact (p = 0,05) and distant (> 10 mm) to the ventricle system (p = 0,038). On the other hand, STS showed higher frequency of GBM location close (≤ 10 mm) to the ventricle system (p = 0,05), a higher incidence of SVZ involvement (p = 0,05) and subependymal spread (p = 0,066). These findings correspond with reports in the literature [10,11] (*) where tumor localization close to the SVZ or ventricle system was connected with decreased survival. Parsa et al. described an increased survival rate of GBM patients with subependymal tumor spread in a collective of patients with disseminated GBM disease [20]. Chaichana et al. [7] found a negative prognostic association between periventricular tumor localization and survival. The main reasons for a less favorable outcome in GBM patients with SVZ involvement are not yet completely understood. Experiments and clinical findings provide evidence that neuronal progenitor cells in the SVZ with a high migratory potential are involved in an aggressive glioblastoma subtype [21]. Another possibility is the close proximity of GBM with ventricle association to a higher density of subcortical fibers and critical neurological tissue than peripheral tumors [22].

Furthermore, surgical resection status during initial treatment is an established predictive factor for survival in GBM patients. Total resection is presumed to be associated with a favorable prognosis [3,12,23]. In our collective of LTS, a higher rate of completely resected patients at the time of initial treatment could be observed (p = 0,002). In contrast a higher rate of STS were diagnosed by biopsy previous to the primary radio- or radio-/chemotherapy (p < 0,001), concluding that complete resection status is prognostic for improved survival in patients with GBM.

There was no significant difference between the ratio of LTS and STS receiving a concomitant chemoradiotherapy with temozolomide. Overall the fraction receiving a combined therapy was high in both groups, with about 70%. Temozolomide-based bimodal therapy is known to increase survival rates in glioblastoma patients [1].

Our study has several limitations due to its retrospective design. This cohort was not consecutively treated. Furthermore patient’s performance status (Karnofsky index), was not documented consistently and could not be evaluated. Poor performance status is known to correlate with worse OS and may lead to major bias for our survival analysis. Resection status as an important covariate may confound our survival analysis with regard to the SVZ, taking into account that LTS had a higher frequency of total resections (22 vs. 7 patients).

Moreover numerous patients with malignant glioma are included in study trials in our department, to evaluate new clinical approaches. In this study, the proportion of GBM patients being treated within a study trial was almost equal in LTS and STS with about 50% each, concluding that treatment in study setups in our department is non-inferior to our standard treatment protocols.

Despite clinical characteristics, molecular markers play a growing role in determining therapeutic response and prognosis in GBM patients [13,14]. Our data show a higher rate of MGMT hypermethylated promotors in GBM LTS than in STS. The patient groups were too small to achieve a significant pronunciation (p = 0,121). These findings support the clinical results implicating MGMT promoter methylation as a positive predictor for therapeutic response to temozolomide and increased survival [8]. It is important to add that neither lacking MGMT-hypermethylation precludes long-term survival nor MGMT-hypermethylation prevents a patient from becoming a STS.

## Conclusions

This study underlines that survival in GBM patients is heterogeneous and influenced by multiple factors. We found a correlation between long-term survival and young age, total resection status, no SVZ contact and localization distant to the ventricle system.

On the other hand, these data suggest an association between short-term survival and higher age, no resection, SVZ involvement, localization close to the ventricle system and subependymal tumor spread.

These prognostic indicators serve to identify high risk groups that should be treated at an early course of the disease with an aggressive regime. Furthermore these findings help to estimate prognosis, define treatment volume in radiotherapy and serve as a foundation for future investigations on LTS and STS.

## Competing interests

The authors declare that they have no competing interests.

## Authors’ contributions

SC and JD treated the patients. SA, LK and SC collected the data. SA and SC evaluated the dataset and performed statistical analysis. TW and TB reviewed the radiological imaging. SA, SC, TB, TW and JD wrote and edited the manuscript. All authors read and approved the manuscript.
